# Assessment of knowledge, attitudes, and practices regarding TB prevention and treatment among college students in Wuhan, central China

**DOI:** 10.3389/ftubr.2025.1587839

**Published:** 2025-08-01

**Authors:** Gang Wu, Chao Quan, Aiping Yu, Li Li, Jun Chen, Yun Yang, Guiyang Wang, Zhouqin Lu, Xiaojun Wang, Qionghong Duan, Haoze Zhang, Jing Hu, Zhengbin Zhang

**Affiliations:** ^1^Department of Tuberculosis Control, Wuhan Pulmonary Hospital (Hubei Branch of National Clinical Research Center for Infectious Diseases), Wuhan, Hubei, China; ^2^Infectious Disease Prevention and Control Department, Dongxihu Centers for Disease Prevention and Control, Wuhan, Hubei, China; ^3^Department of Tuberculosis Control, Hongshan Centers for Disease Prevention and Control, Wuhan, Hubei, China; ^4^Occupational Disease Prevention and Control Department, Center for Disease Control and Prevention of Yangtze River Navigation Administration, Wuhan, Hubei, China

**Keywords:** tuberculosis, students, college, KAP, influencing factor

## Abstract

**Background:**

Tuberculosis (TB) is recognized as a serious public health concern in many developing countries. As the new main force and inheritors of national health construction, college students bear important social responsibilities and missions of practicing TB prevention and control, volunteer mobilization, and health concept dissemination. Improving college students' health literacy is crucial to preventing and treating infectious diseases. This study aims to assess college students' knowledge, attitudes, and practices (KAP) about TB prevention and treatment in Wuhan, China.

**Methods:**

A descriptive cross-sectional study survey among college students was conducted from May to June 2022 at 22 colleges using the judgment sampling method in Wuhan. KAP regarding TB was recorded and assessed using an online questionnaire platform. The status quo of KAP regarding TB prevention and treatment was statistically described, and the formation of students' TB prevention and treatment behavior was analyzed using single-factor and multifactor logistic regression.

**Results:**

A total of 15,290 college students completed the questionnaire, and 15,272 valid questionnaires were collected. The total awareness rate of knowledge, attitudes, and practices of TB prevention and treatment was 79.69%. Among them, the awareness rate of key knowledge was 79.28%, and the passing rate was 92.46%. The holding rate of correct attitudes was 89.69%, and the passing rate was 90.56%. The formation rate of correct behaviors was 72.11%, and the passing rate was 96.62%. A single-factor analysis showed that gender, grade, parents' education level, contact history of TB patients, and passing rate of key knowledge were statistically different in the formation of prevention and treatment behaviors. Multivariate logistic regression analysis showed that for females (odds ratio [OR] = 1.86, 95% CI [1.50, 2.30], *p* < 0.01), students with father's educational background of primary school (OR = 2.94, 95%CI [1.62, 5.33], *p* < 0.01), junior school and equivalent (OR = 2.94, 95%CI [1.62, 5.33], *p* < 0.01), high school and equivalent (OR = 3.05, 95%CI [1.70, 5.46], *p* < 0.01), and diploma or undergraduate (OR = 3.24, 95% CI [1.73, 6.09], *p* < 0.01); no history of TB (OR = 3.32, 95% CI [1.49, 7.41], *p* < 0.01); a passing key knowledge score (OR = 9.91, 95% CI [8.11, 12.11], *p* < 0.01); and a passing attitude score (OR = 7.35, 95% CI [6.01, 8.99], *p* < 0.01) were likely to form TB prevention and treatment behaviors.

**Conclusion:**

School health promotion should not only focus on improving students' personal knowledge and skills of TB prevention and treatment but also emphasize the level of family participation, strengthen the comprehensive mobilization action of schools, formulate policies to support health action, create a supportive social environment, and radiate the health education effect of students to the whole society.

## 1 Introduction

Tuberculosis (TB) remains a major public health problem in China (1–3); according to the World Health Organization, in 2024, an estimated 741,000 new TB cases occurred in China, accounting for 6.8% of the total number of TB cases worldwide, and 25,000 people died from TB (4). Research evidence shows that improving people's knowledge and attitudes is conducive to forming healthy behaviors and achieving TB prevention and treatment goals (5, 6), especially for college students. As we know, college students' health education, which affects people's life cycle, has extraordinary significance in not only teaching them health knowledge but also encouraging them to convey health information to other groups of people and promoting the educational effect to radiate to society. However, many researchers focus on improving individual health literacy through school education or expert training (7, 8); little attention is paid to the influence of family and parental participation or social environment support simultaneously. Doing more research is quite necessary for a comprehensive understanding of the factors that affect students' knowledge, attitudes, and practices (KAP) regarding TB prevention and control.

Wuhan is a sub-provincial city in central China, located in the eastern part of the Jianghan Plain in the middle reaches of the Yangtze River. It has 14 districts under its jurisdiction, with a total area of 8,569.15 square kilometers and a permanent population of 13.77 million, including more than 1.3 million college students attending 96 universities or colleges (9). There are nearly 10 school TB outbreaks in Wuhan every year (10, 11), and more than 500 students with TB are found, accounting for about one-eighth of the registered TB patients in Wuhan. Therefore, the government health administration has for some time attached great importance to preventing and treating TB in universities or colleges, especially college students' health promotion work.

This study aims to assess the KAP of TB prevention and treatment among college students and explore the influencing factors of assessment outcomes so as to carry out more comprehensive health promotion work on TB prevention and treatment among students.

## 2 Materials and methods

### 2.1 Study site and study design

This study was conducted in 22 colleges or universities, accounting for about one-fourth of the universities or colleges in Wuhan. The Hongshan District Center for Disease Control and Prevention assisted the Wuhan TB prevention and treatment institute in implementing this study. We selected 15,290 college students from 22 universities or colleges using judgment sampling according to the school type (16 national public universities and 6 private universities of enterprises, institutions or social organizations), geographical location, total number of students (total number of students in school should reach over 8,000), TB epidemic situation (more TB patients have been found in the last 3 years), and the implementation of daily prevention and treatment measures (the implementation of TB prevention and treatment measures is relatively poor) in last 3 years. Included in the study were individuals who were at least 17 years old; were freshmen, sophomores, juniors, seniors, and above; and were selected from each grade group according to their majors. Each student answered an online survey questionnaire using a scanning app on their mobile phone. The questionnaire was made using Questionnaire Star, a data collection, analysis, and management online survey platform developed by Ranxing Information Technology Co., Ltd. in Changsha, China. College students from different grades, departments, and majors were required to anonymously fill out the questionnaire survey by forwarding the Questionnaire Star QR code through online platforms, such as WeChat and QQ groups, based on the principle of informed consent, through the school doctor or the student affairs office. When the survey was completed, all data were collected at the backend by Questionnaire Star for completeness and logical checks. The questionnaire's internal consistency reliability was assessed by using Cronbach's alpha test, yielding a value of 0.71, indicating good reliability.

### 2.2 Data collection

The questionnaire included sociodemographic characteristics and three dimensions of key KAP, as shown in Table 1. Sociodemographic characteristics included 14 items, such as gender, age, major, nationality, registered residence, parents' educational background, and contact history with TB patients and history of TB. The knowledge of TB prevention and treatment included five items: the etiology of TB, the TB transmission route, common suspicious symptoms of TB, where to receive an examination and treatment, and whether TB can be cured. Beliefs and attitudes included four items: whether to master prevention and treatment knowledge, whether to carry out health education, whether to take the initiative to report to the school when sick, and whether to discriminate against TB patients. Prevention and treatment behaviors included five items: seeking medical service promptly if suspected of illness, suspending schooling due to TB, spitting etiquette, and actively learning disease prevention knowledge and skills.

**Table 1:** Knowledge, attitudes and practices of tuberculosis prevention and treatment among college students.

**Items**	**No. of students who answered correctly**	**The proportion of students who answered correctly**
**Key information of tuberculosis prevention and control**		
What is the etiology of tuberculosis?	7,838	51.32%
What are the main transmission routes of pulmonary tuberculosis?	13,618	89.17%
What are the common suspicious symptoms of pulmonary tuberculosis?	13,794	90.32%
Where to receive examination and treatment for suspected tuberculosis?	14,688	96.18%
Can tuberculosis be cured by adhering to regular treatment?	10,597	69.39%
**Attitudes toward tuberculosis prevention and treatment**		
Whether college students need to master tuberculosis prevention knowledge actively	14,810	96.97%
Should schools regularly carry out health education activities on tuberculosis prevention and treatment?	14,377	94.14%
Should students report suspected symptoms of tuberculosis or be diagnosed with tuberculosis to their teachers proactively and not conceal their illness?	12,918	84.59%
Should we exclude or discriminate against tuberculosis patients?	12,682	83.04%
**Tuberculosis prevention and treatment practices**		
What should we do if we suspect ourselves of having tuberculosis?	14,688	96.18%
Can you implement the policy of taking leave and being treated in isolation at home after tuberculosis is suspected or diagnosed?	15,066	98.65%
Will you persuade and stop students around you who spit everywhere?	6,095	39.91%
Do you use elbows/tissues to cover your mouth and nose when coughing or coughing up phlegm?	9,379	61.41%
Do you actively learn about tuberculosis prevention knowledge?	9,836	64.41%

### 2.3 Scoring criteria

Knowledge awareness was scored as 1 point for each correct answer and 0 points for each wrong answer, for a maximum score of 5 points. A score of ≥3 points is considered passing. For scoring attitude, according to the positive attitude rating, it is divided into necessary (*agree*), indifferent (*neutral*), and unnecessary (*disagree*), with scores of 2, 1, and 0, respectively. The highest score was 8 points, and a score of ≥5 points is considered passing. The scoring criteria for the formation of prevention and treatment behaviors are divided into five levels: proactive (*always*), relatively proactive (*often*), average (*sometimes*), indifferent (*occasionally*), and unwilling (*never*), with scores of 4, 3, 2, 1, and 0, respectively. The maximum score was 20 points, and a score of ≥12 is considered passing.

### 2.4 Statistical formula

The awareness rate of a single item was determined by the number of participants who correctly answered a certain item divided by the total number of survey respondents and multiplied by 100%. The total awareness rate of key knowledge was calculated as the sum of correct answers to key information items by each survey participant divided by the sum of answers to key information items by all survey respondents and multiplied by 100%. The total holding rate of correct attitudes was determined by the sum of correct attitude information items answered by each survey participant divided by the sum of attitude information items answered by all survey respondents and multiplied by 100%. The total formation rate of correct behaviors was determined by the sum of the number of correct behavior information items answered by each survey participant divided by the sum of the number of behavior information items answered by all survey respondents and multiplied 100%. The passing rate of knowledge awareness was computed as the number of participants who passed the knowledge awareness score divided by the total number of survey respondents multiplied by 100%. The passing rate of positive attitudes was determined by the number of participants who passed the correct attitude answer score divided by the total number of survey respondents and multiplied by 100%. The passing rate of positive behaviors formation was calculated as the number of participants who pass the behaviors formation answer score divided by the total number of survey respondents and multiplied by 100%.

### 2.5 Quality control

Before the formal investigation, a pre-survey of 150 questionnaires was conducted, and it was determined that at least 5 min were needed to complete the questionnaire. Then the survey questionnaire was modified and improved, and a reliability analysis of the pre-survey results was conducted. The Centers for Disease Control and Prevention in all 14 districts received unified training. They emphasized preventive measures through the school TB prevention and treatment QQ working group in Wuhan, used unified guiding language, answered questions from teachers and students, and avoided using suggestive language to ensure the quality of questionnaires.

### 2.6 Data management and statistical analysis

The original data were imported into the SPSS 25.0 database in the form of an Excel worksheet by using the “analysis download” function on Questionnaire Star. The concentration and dispersion trends of student age were described by the median of the interquartile range (IQR). The counting data were described by the frequency (*n*) and percentage. The comparison of the passing rate of KAP about TB prevention and treatment was conducted using a χ^2^ test. The influencing factors of practice behaviors were analyzed by the multifactor unconditional logistic regression analysis. The relationship between each factor and tuberculosis prevention and treatment behaviors was estimated by the odds ratio (OR) and 95% confidence interval (CI) and a correlation analysis using the Spearman test. Using a two-sided test, *p* < 0.05 is considered statistically significant.

### 2.7 Ethics statement

Ethical approval for this study was granted by the Wuhan Pulmonary Hospital Medical Ethics Committee (Protocol Number: 2022-58). Before the formal investigation, informed consent was presented in the online survey questionnaire, and the next step of the questionnaire could only be filled out with the consent of students aged 18 and older. For students younger than 18, it was indicated that they should seek their parents' consent by phone before participating in the online survey.

## 3 Results

### 3.1 Study population and content

From May 10 to June 25, 2022, non-probability sampling (judgment sampling method) was used to select different departments and majors from 22 universities in Wuhan. Freshmen, sophomores, juniors, seniors, and students above were selected as survey subjects through stratified sampling. At least 200 students were surveyed in each selected school, including at least 50 students in each grade. In total, 15,290 questionnaires were collected, including 15,272 valid ones (12 questionnaires with a response time of < 5 min and 6 questionnaires that selected a specific answer to every question or fell into trap questions were excluded), with an effective response rate of 99.89%. Among participants, 7,666 were males and 7,606 were females, with a gender ratio of 1.08:1. The age range was 17–32 years old, with a median *M* (P25, P75) of 20 (19, 21) years old. The majority of the population was Han Chinese, accounting for 93.71% (14,312/15,272). There were 9,440 people with registered residence in cities and 5,832 people in rural areas, with a ratio of urban-to- rural population was 1.62:1. Among them, freshmen accounted for 43.71% (6,676/15,272); sophomores, 31.59% (4,825/15,272); juniors, 19.25% (2,940/15,272); and seniors and above, 5.44% (831/15,272).

The content of the questionnaires includes demographic sociological characteristics and three dimensions of key KAP behavior, as shown in Table 1. Sociological characteristics included 14 items, including gender, age, major, nationality, registered residence, parents' educational backgrounds, contact history with TB patients, history of TB, and knowledge of TB prevention and treatment. There were five, four, and five questions, respectively, on KAP behavior problems regarding TB.

### 3.2 KAP

#### 3.2.1 Knowledge awareness of TB prevention and treatment among college students

The total awareness rate of KAP regarding TB prevention and treatment among college students was 79.69% (170,386/213,808). The awareness rate of key knowledge about pulmonary TB was 79.28% (60,535/76,360), with the lowest awareness rate of the nature of pulmonary TB disease being only 51.32% and the highest awareness rate of correct practices for suspected pulmonary TB, reaching 96.18%. The correct attitude rate of TB prevention and treatment was 89.69% (54,787/61,088), and the attitude accuracy rate of the four items was more than 80%, with the highest item being “college students need to master tuberculosis prevention and treatment knowledge” (96.97%). The formation rate of correct behaviors in prevention and treatment was 72.11% (55,064/76,360), while the formation rate of behaviors without spitting around was only 39.91%. Students suspected of having or diagnosed with TB had the highest proportion of prevention and treatment behaviors, such as staying away from school, reaching 98.65%, as shown in Table 1. In addition, the correlation analysis between KAP shows that knowledge scores are positively correlated with attitude scores and behavior scores (*r*s = 0.252 and 0.387, respectively), the same as attitude scores with behavior scores (*r* = 0.373), but the correlation is relatively weak.

#### 3.2.2 Knowledge of TB prevention and treatment among students with different demographic characteristics

The passing rate of college students' awareness of the key knowledge of tuberculosis prevention and treatment was 92.46%. The awareness rate of key knowledge among college students with different gender, parental education level, patient contact history, and medical history was statistically significant (χ^2^ = 95.31, 71.71, 77.39, 65.81, and 31.05, respectively, and *p*-values were all < 0.05), while the awareness rate of key knowledge among college students of different ethnic groups, registered residence, majors, and grades was not significantly different (χ^2^ = 0.01, 0.17, 0.37, and 2.60, respectively, and *p*-values were all >0.05), as shown in Table 2.

**Table 2:** Passing rates of key knowledge of tuberculosis prevention and treatment among college students with different demographic characteristics.

**Group**	**Key knowledge**		**Sum**	**χ^2^**	***p*-value**
	**No. of passing students**	**No. of failed students**			
**Gender**				**95.31**	<**0.01**
Male	6,929 (90.39%)	737 (9.61%)	7,666		
Female	7,192 (94.46%)	414 (5.44%)	7,606		
**Nationality**				**0.01**	**0.97**
Han Chinese	13,233 (92.46%)	1,079 (7.54%)	14,312		
Non-Han Chinese	888 (92.50%)	72 (7.50%)	960		
**Registered residence**				**0.17**	**0.68**
Countryside	8,735 (92.53%)	705 (7.47%)	9,440		
City	5,386 (92.35%)	446 (7.65%)	5,832		
**Major**				**0.37**	**0.55**
Medicine	812 (91.13%)	79 (8.87%)	891		
Non-medicine	13,018 (0.52%)	1,363 (9.48%)	14,381		
**Grade**				**2.60**	**0.46**
Freshman	6,196 (92.81%)	480 (7.18%)	6,676		
Sophomore	4,449 (92.21%)	376 (7.79%)	4,825		
Junior	2,715 (92.35%)	225 (7.65%)	2,940		
Senior and above	761 (91.58%)	70 (8.42%)	831		
**Father's educational level**				**71.71**	<**0.01**
Illiterate or semiliterate	249 (86.46%)	39 (13.54%)	288		
Primary school	2,458 (92.79%)	191 (7.21%)	2,649		
Junior high school or equivalent education	5,441 (92.91%)	415 (7.09%)	5,856		
High school and equivalent education	3,196 (93.10%)	237 (6.90%)	3,433		
Diploma or undergraduate	2,642 (91.96%)	23 (8.04%)	2,873		
Postgraduate	135 (78.03%)	38 (21.97%)	173		
**Mother's educational level**				**77.39**	<**0.01**
Illiterate or semiliterate	467 (87.78%)	65 (12.22%)	532		
Primary school	3,479 (92.48%)	283 (7.52%)	3,762		
Junior high school or equivalent education	5,337 (93.24%)	387 (6.76%)	5,724		
High school and equivalent education	2,774 (92.00%)	241 (8.00%)	3,015		
Diploma or undergraduate	1,975 (93.16%)	145 (6.84%)	2,120		
Postgraduate	89 (74.79%)	30 (25.21%)	119		
**Contact history of TB patients**				**65.81**	<**0.01**
Yes	550 (95.16%)	28 (4.84%)	578		
No	10,826 (93.22%)	787 (6.78%)	11,613		
Unclear	2,745 (89.09%)	336 (10.90%)	3,081		
**History of TB**				**31.05**	<**0.01**
Yes	71 (77.17%)	21 (22.83%)	92		
No	14,050 (92.56%)	1,130 (7.44%)	15,180		

#### 3.2.3 Attitudes toward TB prevention and treatment among students with different demographic characteristics

The positive passing rate of college students' attitudes toward TB prevention and treatment in Wuhan was 90.56%, among which there was a statistically significant difference in the passing rate of students' attitudes according to gender, registered residence, grade, parental education, patient contact history, and tuberculosis history (χ^2^ = 74.71, 5.54, 14.15, 83.60, 60.23, 19.05, and 11.09, respectively; all *p*-values < 0.05). There was no significant difference in the passing rate of students' attitudes by ethnicity and major (χ^2^ = 1.14 and 0.33, respectively; all *p*-values > 0.05), as shown in Table 3.

**Table 3:** Passing rates of tuberculosis prevention and treatment attitudes among college students with different demographic characteristics

**Group**	**Attitudes**		**Sum**	**χ^2^**	***p*-value**
	**No. of passing students**	**No. of failed students**			
**Gender**				**74.71**	<**0.01**
Male	6,786 (88.52%)	880 (11.48%)	7,666		
Female	7,044 (92.61%)	562 (7.39%)	7,606		
**Nationality**				**1.14**	**0.29**
Han Chinese	12,970 (90.62%)	1,342 (9.38%)	14,312		
Non-Han Chinese	860 (89.58%)	100 (10.42%)	960		
**Registered residence**				**5.54**	**0.02**
Countryside	8,590 (91.00%)	850 (9.00%)	9,440		
City	5,240 (89.85%)	592 (10.15%)	5,832		
**Major**				**0.33**	**0.57**
Medicine	802 (90.01%)	89 (9.99%)	891		
Non-medicine	13,028 (90.59%)	1,353 (9.41%)	14,381		
**Grade**				**14.15**	<**0.01**
Freshman	6,466 (96.85%)	210 (3.15%)	6,676		
Sophomore	4,335 (89.84%)	490 (10.16%)	4,825		
Junior	2,653 (90.24%)	287 (9.76%)	2,940		
Senior and above	735 (88.45%)	96 (11.55%)	831		
**Father's educational level**				**83.60**	<**0.01**
Illiterate or semi literate	237 (82.29%)	51 (17.71%)	288		
Primary school	2,408 (90.90%)	241 (9.10%)	2,649		
Junior high school or equivalent education	5,358 (91.50%)	498 (8.50%)	5,856		
High school and equivalent education	3,119 (90.85%)	314 (9.15%)	3,433		
Diploma or undergraduate	2,579 (89.77%)	294 (10.23%)	2,873		
Postgraduate	129 (74.57%)	44 (25.43%)	173		
**Mother's educational level**				**60.23**	<**0.01**
Illiterate or semiliterate	462 (86.84%)	70 (13.16%)	532		
Primary school	3,412 (90.70%)	350 (9.30%)	3,762		
Junior high school or equivalent education	5,224 (91.26%)	500 (8.74%)	5,724		
High school and equivalent education	2,741 (90.91%)	274 (9.09%)	3,015		
Diploma or undergraduate	1,905 (89.86%)	215 (10.14%)	2,120		
Postgraduate	86 (72.27%)	33 (27.73%)	119		
**Contact history of TB patients**				**19.05**	<**0.01**
Yes	524 (90.66%)	54 (9.34%)	578		
No	10,579 (91.97%)	1,034 (8.90%)	11,613		
Unclear	2,727 (88.51%)	354 (11.49%)	3,081		
**History of TB**				**11.09**	<**0.01**
Yes	74 (80.43%)	18 (19.57%)			
No	13,756 (90.62%)	1,424 (9.38%)			

TB, tuberculosis.

#### 3.2.4 Formation of practice behaviors about TB prevention and treatment among students with different demographic characteristics

The positive formation passing rate of college students' TB prevention and treatment behaviors was 96.62%. According to students' gender, grade, parents' educational levels, contact history with TB patients, history of TB, and passing rates of key knowledge and attitudes, there were statistical differences in the distribution of practice behaviors pass (χ^2^ = 91.83, 10.28, 137.46, 103.49, 16.50, 47.37, 1,992.54, and 1,138.22, respectively; all *p*-values < 0.05). There was no significant difference in the passing rate of students' attitudes by nationality, registered residence, and majors (χ^2^ = 1.05, 1.45, and 1.93, respectively; all *p*-values >0.05), as shown in Table 4.

**Table 4:** Passing rates of tuberculosis prevention and treatment behaviors among college students with different demographic characteristics.

**Group**	**Practic**		**Sum**	**χ^2^**	***p*-value**
	**No. of passing students**	**No. of failed students**			
**Gender**				**91.83**	<**0.01**
Male	7,300 (95.23%)	366 (4.77%)	7,666		
Female	7,456 (98.03%)	150 (1.97%)	7,606		
**Nationality**				**1.05**	**0.31**
Han Chinese	13,834 (96.67%)	478 (3.34%)	14,312		
Non-Han Chinese	922 (6.248%)	38 (7.364%)	960		
**Registered residence**				**1.45**	**0.23**
Countryside	9,108 (96.48%)	332 (3.52%)	9,440		
City	5,648 (96.84%)	184 (3.16%)	5,832		
**Major**				**1.93**	**0.17**
Medicine	868 (97.42%)	23 (2.58%)	891		
Non-medicine	13,797 (96.55%)	493 (3.45%)	14,290		
**Grade**				**10.28**	**0.02**
Freshman	6,466 (96.85%)	210 (3.15%)	6,676		
Sophomore	4,647 (96.31%)	178 (3.69%)	4,825		
Junior	2,853 (97.04%)	87 (2.96%)	2,940		
Senior and above	790 (95.07%)	41 (4.93%)	831		
**Father's educational level**				**137.46**	<**0.01**
Illiterate or semiliterate	252 (87.50%)	36 (12.50%)	288		
Primary school	2,563 (96.75%)	86 (3.25%)	2,649		
Junior high school or equivalent education	5,688 (97.13%)	168 (2.87%)	5,856		
High school and equivalent education	3,327 (96.91%)	106 (3.09%)	3,433		
Diploma or undergraduate	2,777 (96.66%)	96 (3.34%)	2,873		
Postgraduate	149 (86.13%)	24 (13.87%)	173		
**Mother's educational level**				**103.49**	<**0.01**
Illiterate or semiliterate	490 (92.11%)	42 (7.89%)	532		
Primary school	3,634 (96.60%)	128 (3.40%)	3,762		
Junior high school or equivalent education	5,578 (97.45%)	146 (2.55%)	5,724		
High school and equivalent education	2,911 (96.55%)	104 (3.45%)	3,015		
Diploma or undergraduate	2,043 (96.37%)	77 (3.63%)	2,120		
Postgraduate	100 (84.03%)	19 (15.97%)	119		
**Contact history of TB patients**				**16.50**	<**0.01**
Yes	563 (97.40%)	15 (2.60%)	578		
No	11,252 (96.89%)	361 (3.11%)	11,613		
Unclear	2,941 (95.46%)	140 (4.54%)	3,081		
**History of TB**				**47.37**	<**0.01**
Yes	77 (83.70%)	15 (16.30%)	92		
No	14,679 (96.70%)	501 (3.30%)	15,180		
**Key knowledge awareness meets the standard**				**1992.54**	<**0.01**
Pass	13,907 (98.48%)	214 (1.52%)	14,121		
Fail	849 (73.76%)	302 (26.24%)	1,151		
**Attitudes meet the standard**				**1138.22**	<**0.01**
Pass	13,583 (98.21%)	247 (1.79%)	13,830		
Fail	1,173 (81.35%)	269 (18.65%)	1,442		

TB, tuberculosis.

#### 3.2.5 Analysis on the influencing factors of college students' TB prevention and treatment behaviors

We took whether the behavior scores of college students pass as the dependent variable (fail = 0, pass = 1), the factors (gender, grade, parents' education levels, contact history with TB patients, TB history, and knowledge and attitudes meet the standards), with *p* < 0.05, in the single-factor analysis of prevention and treatment behaviors were included in the independent variable for logistic regression analysis. The results showed that the being female, having a father with a high education level, having no history of TB, and having a high knowledge awareness rate and correct attitudes rate were more likely to promote the formation of healthy behaviors (*p* < 0.05), as shown in Table 5.

**Table 5:** Multivariate analysis on the influencing factors of college students' tuberculosis prevention and treatment behaviors.

**variables**	**No. of passing students**	**No. of failed students**	**COR [95% CI]**	***p-*value**	**AOR [95% CI]**	***p-*value**
**Gender**			**2.49 [2.06, 3.02]**	<**0.01**		
Male	7,300	366				1
Female	7,456	150			1.86 [1.50, 2.30]	< 0.01
**Grade**			**1.07 [0.98, 1.18]**	**0.02**		
Freshman	6,466	210				1
Sophomore	4,647	178			0.95 [0.75, 1.19]	0.57
Junior	2,853	87			1.09 [0.82, 1.44]	0.63
Senior and above	790	41			0.81 [0.54, 1.23]	0.32
**Father's educational level**			**[0.93, 1.10]**	<**0.01**		
Illiterate or semiliterate	252	36				1
Primary school	2,563	86			2.94 [1.62, 5.33]	< 0.01
Junior school or equivalent education	5,688	168			3.05 [1.70, 5.46]	< 0.01
High school and equivalent education	3,327	106			3.17 [1.72, 5.87]	< 0.01
Diploma or undergraduate	2,777	96			3.24 [1.73, 6.09]	< 0.01
Postgraduate	149	24			2.18 [0.81, 5.85]	0.12
**Mother's educational level**			**0.99 [0.91, 1.07]**	<**0.01**		
Illiterate or semiliterate	490	42				1
Primary school	3,634	128			1.06 [0.62, 1.80]	0.84
Junior high school or equivalent education	5,578	146			1.33 [0.78, 2.27]	0.29
High school and equivalent education	2,911	104			0.94 [0.53, 1.65]	0.82
Diploma or undergraduate	2,043	77			0.88 [0.48, 1.60]	0.67
Postgraduate	100	19			0.81 [0.28, 2.35]	0.70
**Contact history of TB patients**			**0.69 [0.58, 0.83]**	<**0.01**		
Yes	563	15				1
No	11,252	361			0.67 [0.35, 1.27]	0.22
Unclear	2,941	140			0.58 [0.30, 1.13]	0.11
**History of TB**			**5.71 [3.26, 10.00]**	<**0.01**	**–**	
Yes	77	15				1
No	14,679	501			3.32 [1.49, 7.41]	< 0.01
**Key knowledge awareness meets the standard**			**14.99 [12.42, 18.08]**	<**0.01**		
Fail	1,610	334				1
Pass	13,146	182			9.91 [8.11, 12.11]	< 0.01
**Attitudes meet the standard**			**12.61 [10.51, 15.14]**	<**0.01**		
Fail	1,173	269				1
Pass	13,583	247			7.35 [6.01, 8.99]	< 0.01

COR, crude odds ratio; AOR, adjusted odds ratio, TB, tuberculosis.

## 4 Discussion

KAP has been the main educational intervention strategy for global TB control, guiding people to acquire relevant health knowledge and skills through learning and gradually establish healthy beliefs and attitudes (12–14) to develop correct healthy behaviors. In our study, we present the KAP on TB prevention, transmission, and control among college students in Wuhan. Understanding students' awareness and practice of TB prevention and treatment may be important for expanding the volunteer team and adjusting the prevention and treatment strategies because college students' participation is an important part of implementing the TB plan.

### 4.1 Knowledge assessment

The results of this study showed that the total passing rate of KAP of college students about TB prevention and treatment in Wuhan was 79.69%. Among them, the awareness rate of key knowledge was 79.28%, which does not yet reach the 85% target requirement of the National Health Commission of China (15). Nearly half of the students did not know that pulmonary TB is a chronic infectious disease caused by *Mycobacterium tuberculosis*, and nearly 30% of the students did not know that regular treatment of pulmonary TB could be cured, indicating that college students' understanding of the key information was not comprehensive and in-depth (16, 17) or that the interpretation of TB diagnosis and treatment knowledge propaganda from the health administration department was not detailed and clear enough. We found a statistically significant difference in the passing rate of key knowledge awareness according to gender, maternal educational level, contact history with TB patients, and history of pulmonary TB. Female students had a better grasp of key knowledge than male students, which might be related to the fact that similar to other infectious diseases, females pay more attention to health and TB knowledge (18). Students whose parents' educational levels were in middle primary school and undergraduate students and below had a better grasp of key knowledge than illiterate students, graduate and above students, and students whose parents' educational levels were too high or low have relatively poor knowledge of TB. This may be because parents with higher educational levels had better family economic incomes, superior family living and working environments, and fewer TB patients around them, weakening their awareness of TB prevention and control (19, 20), while parents with lower educational levels had limited access to information about infectious diseases due to their lower cultural literacy and limited health literacy and education, lowering their ability to inform their children. Students with a history of TB exposure had a better grasp of key knowledge than students without exposure. Students with a history of TB exposure might have experienced close contact screening and pay more attention to learning TB prevention and treatment knowledge. There was no significant difference in the passing rate of students' key knowledge awareness by nationality, registered residence, major, and grade. This is inconsistent with other similar studies at home and abroad. It may be that during the COVID-19 pandemic period, due to the strict isolation and control measures taken nationwide, people paid more attention to respiratory infectious diseases and that students received more convenient knowledge and information and took more initiative to learn the relevant knowledge in the short term. Therefore, the difference in the awareness rate of TB core knowledge is not very obvious. Of course, this is just a common assumption that needs further investigation and analysis.

### 4.2 Attitudes assessment

The correct rate of college students' holding attitudes about TB prevention and treatment in Wuhan was 90.56%, and the correct rate of the attitudes toward “Should students report suspected symptoms of tuberculosis or be diagnosed with tuberculosis to their teachers proactively and not conceal their illness?” and “Should we exclude or discriminate against tuberculosis patients?” was < 85%. Our survey results show that when it comes to students' academic delays and personal interests, some students' attitudes are often hesitant and irrational. A few students are still afraid of infection and are unwilling to have contact with TB patients who return to school after treatment, which indicates that these students do not understand the epidemic characteristics of TB treatment and transmission, or affected by the COVID-19 pandemic, they fear respiratory infectious diseases and overestimate the harm of TB and the discrimination and prejudice against TB patients that still exist (21, 22). This indicates that some students have a serious separation between their knowledge and their behaviors, and their proactive awareness of prevention and treatment is indifferent. The rate of females holding correct attitudes is significantly higher than that of males, which may be related to females' greater attention to the knowledge about infectious disease, as well as their greater concern for physical health. The holding rate of students' correct attitudes is similar to their knowledge awareness rate. Interestingly, the proportion of students from rural areas and freshmen with correct attitudes toward TB prevention and treatment is higher than that of urban and senior students. College students from rural areas may be more likely to meet and hear about TB patients in a high epidemic environment, which makes them concerned and learn about TB. At the beginning of college, “the first course of health education,” of which respiratory infectious diseases are required content, is a compulsory course for freshmen. Implementing health courses can optimize accessibility, strengthen communication, and stimulate positive learning desire (23), so students are exposed to knowledge about TB prevention and treatment in a short time.

### 4.3 Practices assessment

The positive formation rate of college students' practices about TB prevention and treatment was 96.62%, and the correct rate of “Will you persuade and stop students who spit everywhere around you?” “Do you use elbows/tissues to cover your mouth and nose when coughing or coughing up phlegm?” and “Do you actively learn about tuberculosis prevention knowledge?” was < 85%, the formation of correct practices and attitudes of students with gender, freshman grade, parents' educational backgrounds, contact history with TB patients, and TB medical history are similar, and the correct practices formation of students with qualified knowledge and attitude is higher than that of students without qualified knowledge, which shows that correct knowledge and attitudes have a positive effect on forming correct behavior. Behavior formation analysis shows that college students generally can restrain their own behaviors; when they are attacked or criticized for discouraging others from engaging in unhealthy behaviors, they may find maintaining correct behaviors difficult or evade the responsibility to dissuade others. It can be seen that future publicity work should not only publicize TB prevention and treatment knowledge but also create environmental conditions and social responsibility guidance for the formation of correct behaviors to enable all social personnel to participate in TB prevention and control and play a larger and broader positive chain reaction.

### 4.4 Influence of TB prevention and treatment practice behaviors

The results of single-factor analysis on the passing rate of behaviors about TB prevention and treatment in this study showed that gender, grade, parents' educational levels, contact history with TB patients, TB history, passing status of key knowledge and attitudes, and other factors had statistical differences in the distribution of passing behaviors. Further multivariate logistic regression analysis showed that female students, those with fathers who had a high educational level, those with no history of TB, and those who had a high awareness of key knowledge and positive attitudes were more likely to promote the formation of healthy practice behaviors (*p* < 0.05). Female students with strong health awareness and those who have not suffered from TB are more inclined to take correct behaviors to reduce the risk of TB infection. The Fathers' educational level had a more significant impact on the formation of behaviors than mothers' (24, 25), indicating the crucial and indispensable role of fathers in family education. In addition, the probability of college students achieving a passing practice behaviors score after passing the key knowledge and attitudes is 9.91 times and 7.35 times higher than those who have failed, respectively, which is far higher than other influencing factors included in the analysis. It can be seen that in the KAP model, knowledge and attitudes are the basis and motivation for changing practice behaviors. It is a gradual and complex process from acquiring knowledge and generating belief to forming healthy behaviors (26). More comprehensive knowledge of correct TB beliefs and positive attitudes are conducive to forming good and healthy behaviors. Therefore, it is of great significance to strengthening college students learning about TB prevention and control knowledge and changing their negative and wrong attitudes to form good health behaviors is of great significance. This also applies to other diseases (27, 28).

## 5 Strengths and limitations of the study

In this study, we have collected 15,272 valid questionnaires from 22 universities. The sample of the questionnaire is sufficient, so the survey on college students' KAP of TB prevention and treatment is representative. Our study also has some limitations. First, our method for selecting the schools was not random sampling but purposive sampling, so the survey may have some sampling bias. However, the student selection was based on random stratified sampling. Second, the depth and scope of the questionnaire survey are limited, which may lead to a certain degree of subjectivity and empiricism in the analysis results. Third, family income, medical insurance coverage, personal education experience, learning and living environments, and other conditions may have an impact on the KAP of TB prevention and control, which may need further investigation and more scientific analysis in the future.

## 6 Conclusion

(1) College students in Wuhan have a certain understanding of TB prevention and control, but there is a serious separation between knowledge, practices, and discrimination against TB patients, and nearly half of the college students have not formed correct prevention and treatment behaviors.
(2) Gender, history of pulmonary TB, an awareness of key knowledge about prevention and treatment attitudes and cognition, and parents' educational backgrounds are the main influencing factors on TB prevention and treatment behaviors of college students, the level of parental involvement in families needs to be strengthened, and behavioral intervention measures should be implemented based on students' characteristics.
(3) Schools should pay attention to guiding good infectious disease prevention and treatment behaviors, encouraging students to actively participate in practices, and creating a good campus environment and policy support. Health and education administrative departments should jointly strengthen professional training and technical guidance for schools and even provide funds for conducting health education activities.

## Data Availability

The raw data supporting the conclusions of this article will be made available by the authors, without undue reservation.
